# An Overview of the Potentialities of Antimicrobial Peptides Derived from Natural Sources

**DOI:** 10.3390/antibiotics11111483

**Published:** 2022-10-26

**Authors:** Irene Dini, Margherita-Gabriella De Biasi, Andrea Mancusi

**Affiliations:** 1 Department of Pharmacy, University of Naples Federico II, Via Domenico Montesano 49, 80131 Napoli, Italy; 2 Department of Food Microbiology, Istituto Zooprofilattico Sperimentale del Mezzogiorno, Via Salute 2, 80055 Portici, Italy

**Keywords:** AMPs, food preservation, food shelf-life, active packaging, Gram-positive bacteria, Gram-negative bacteria, antibiotics, antibiotic resistance, innate immune defense molecules, mechanism of action, delivery systems

## Abstract

Antimicrobial peptides (AMPs) are constituents of the innate immune system in every kind of living organism. They can act by disrupting the microbial membrane or without affecting membrane stability. Interest in these small peptides stems from the fear of antibiotics and the emergence of microorganisms resistant to antibiotics. Through membrane or metabolic disruption, they defend an organism against invading bacteria, viruses, protozoa, and fungi. High efficacy and specificity, low drug interaction and toxicity, thermostability, solubility in water, and biological diversity suggest their applications in food, medicine, agriculture, animal husbandry, and aquaculture. Nanocarriers can be used to protect, deliver, and improve their bioavailability effectiveness. High cost of production could limit their use. This review summarizes the natural sources, structures, modes of action, and applications of microbial peptides in the food and pharmaceutical industries. Any restrictions on AMPs’ large-scale production are also taken into consideration.

## 1. Introduction

Antimicrobial peptides (AMPs) are the oldest known innate immune defense molecules. They are abundant in plants, arthropods, microorganisms, and animals [1]. Eukaryotes and prokaryotes synthesize AMPs in ribosomes, fungi, and bacteria, turning them into cytosol [2]. AMPs can have broad-spectrum or specific activity against pathogenic bacteria (both Gram-positive and Gram-negative), viruses, fungi, and other parasites [3]. AMPs differ in length and composition of amino acids [4]. Defensins, puroindolines, snakins, cyclotides, glycine-rich proteins, hevein, α-hairpin, knottin, and lipid transfer proteins are some natural classes of AMPs [5]. Their activity is bound by helical structure, charge, hydrophobicity, and amphipathicity [4]. The food industry employs AMPs as biopreservants and in food packaging (alone or with other antimicrobials and essential oils) to improve product shelf-life [6]. Antimicrobial peptides are considered potential drugs for treating infections caused by microorganisms that are untreatable with antibiotics on the market today [7,8]. They can reduce the development of antimicrobial resistance, affecting multiple low-affinity targets [9]. Some AMPs are subjected to peptide engineering and mutagenesis to make compounds with improved bioactivity and reduced cytotoxicity [10,11]. This review offers an overview of structures, sources, modes of action, and applications of AMPs in the food and pharmaceutical fields.

## 2. Antimicrobial Peptides’ Natural Source

**Figure 1:**
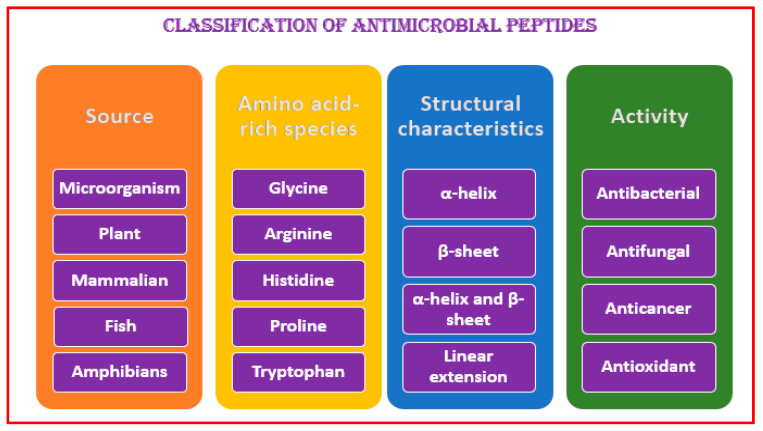
Several ways to classify antimicrobial peptides (AMPs).

Antimicrobial peptides are made by lower and higher organisms responding to pathogenic challenges [12]. AMPs kill the invading pathogens and modulate the innate immune response. They are commonly classified according to their sources, amino-acid-rich species, structural characteristics, and activities [13]. In multicellular organisms and humans, they are localized into specific sites commonly exposed to microbes (i.e., mucosa epithelia and skin) [13] (Figure 1).

### 2.1. Viral AMPs

Some phage proteins, including lysins, depolymerases, virion-associated peptidoglycan hydrolases (VAPGHs), and holins, show antibacterial activity [14]. They are defined as “enzybiotics” to indicate their use as antibacterial materials as alternatives to standard antibiotics [15]. The two types of phage AMPs are known as phage-encoded lytic factors and phage-tail complexes [16].

Phage lysines (size range from 25 to 40 kDa) are peptidoglycan-hydrolyzing enzymes [17], which can hydrolyze the microbial cell wall, permitting bacteriophage progeny release [16]. Lysins have rapid bactericidal activity (against Gram-positive and Gram-negative bacteria) and other desirable characteristics, such as synergy with cell-wall-reducing antibiotics, anti-biofilm action, heat stability up to ~50 °C, and the possibility of lyophilization [18,19,20]. Peptidoglycan hydrolases (VAPGHs), encoded mainly by double-stranded DNA phages, have high thermal stability. They infect Gram-positive and Gram-negative bacteria. VAPGHs have a *C*-terminal cell-wall-binding domain, which can link them to receptors on the bacterial cell surface. They inject genetic materials into bacterial cells after partially and locally damaging bacterial cell wall peptidoglycans [21]. They can be classified into three categories: glycosidases that cut glycosidic bonds in the peptidoglycan chain, amidases that cut amide bonds (between N-acetylmuramic acid lactyl and stem peptide l-alanines), and endopeptidases that cleave peptide bonds within either the stem peptide or cross-link [22].

Phage-tail-like AMPs are high-molecular-weight cylindrical peptides with a structure like a phage tail [23,24]. They can be classified into two classes: R-type (related to Myoviridae phage tails) and F-type (related to Siphoviridae phage tails) [23].

R-type phage-tail-like bacteriocins are nonflexible and have tubes surrounded by contractile sheaths [25]. They initially make a channel in the cell membrane and successively drive their internal core into the cell. This process determines rapid cell death by decoupling cellular ion gradients [23], interfering with oxygen uptake, and affecting macromolecule synthesis [26].

F-type phage-tail-like bacteriocins are flexible and noncontractile [25]. They act similarly to R-type bacteriocins [27].

### 2.2. Bacterial AMPs

#### 2.2.1. AMPs Made by Gram-Positive Bacteria

**Figure 2:**
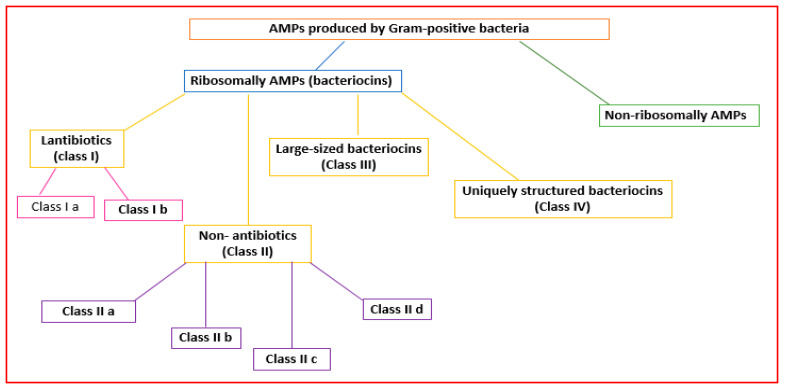
Classification of the AMPs produced by Gram-positive bacteria.

Gram-positive bacteria can produce AMPs in ribosomes (ribosomal AMPs) or enzymatically (non-ribosomal AMPs) [28,29] (Figure 2). Twenty-sixty amino acids (hydrophobic and cationic) can make up ribosomally synthesized bacterial AMPs (bacteriocins) [30].

Bacteriocins can be classified into bacteriocins produced by Gram-positive and Gram-negative bacteria [31].

#### 2.2.2. AMPs Made by Gram-Positive Bacteria

Gram-positive organisms make bacteriocins that can be grouped into lantibiotics (class I), non-lantibiotics (class II), large-sized bacteriocins (class III), and uniquely structured bacteriocins (class IV) [32] (Figure 2).

Lantibiotics are active against primarily Gram-positive bacteria [32]. They are small peptides (<5 kDa; 19–50 amino acids) that are stable to heat, pH, and proteolysis [33]. Lantibiotics can be subdivided into subclasses Ia and Ib (Figure 2).

Subclass Ia lantibiotics form pores in bacterial membranes that determine cellular death [34].

Subclass Ib lantibiotics are inflexible peptides that decrease the activity of bacteria crucial enzymes [32].

Class II AMPs (non-lanthionine-containing bacteriocins) are small (<10 kDa) and heat-stable peptides that can form pores in the bacterial membrane. They can be grouped into four subclasses [35].

Subclass IIa consists of disulfide linear peptides with similar amino acid sequences that permeabilize the cell membrane, showing significant antilisterial activity [36].

Subclass IIb bacteriocins increase the permeability of the bacterial cell membrane to specific small molecules [37]. They contain two peptide subunits (α and β) [37].

Subclass IIc bacteriocins permeabilize the microbial membrane, dissipate the membrane potential, and cause cell death [38]. They comprise small, cyclic peptides whose *C*- and *N*-terminals are covalently linked [39].

Subclass IId comprises the remaining non-characterized bacteriocins in class II [32].

Class III bacteriocins (bacteriolysins) [35] are large (>30 kDa), heat-labile peptides [32] (Figure 2).

Class IV AMPs containing lipids or carbohydrates are susceptible to lipolytic and glycolytic enzymes [40] (Figure 2).

Non-ribosomally synthesized AMPs are made from peptide synthetases produced by Gram-positive and Gram-negative bacteria [9].

Bacteriocins can decrease food spoilage [31].

#### 2.2.3. AMPs Made by Gram-Negative Bacteria

**Figure 3:**
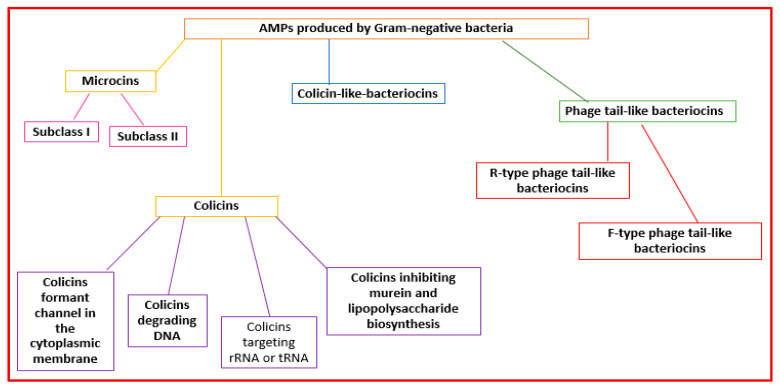
Classification of the AMPs produced by Gram-negative bacteria.

Gram-negative organisms make bacteriocins that can be grouped into microcins, colicins, colicin-like bacteriocins, and phage-tail-like bacteriocins [41] (Figure 3).

Microcins are made by *Enterobacteriaceae*. Microcins interact with some cellular targets. They can format pores that determine membrane disruption [23] or decrease the functionality of enzymes (the ATP synthase complex, DNA gyrase, RNA polymerase, and aspartyl-t RNA synthetase) [32]. They are grouped into two subclasses: subclass I (molecular weight lower than 5 kDa) and subclass II (molecular weight ranging from 5 to 10 kDa) [42,43] (Figure 3).

Colicins (MW > 10 kDa) are made mainly by *Enterobacteriaceae* (mainly *E. coli*) [44]. They can form pores in the cell wall or degrade bacteria nucleic acid structures (RNAses, DNAses, or *t*RNAses) [32]. Colicins can be grouped into four subclasses: colicins forming channels in the cytoplasmic membrane, colicins degrading DNA, colicins targeting rRNA or tRNA, and colicins inhibiting murein and lipopolysaccharide biosynthesis [45,46] (Figure 3).

Colicin-like bacteriocins are made by the *Klebsiella genus* (klebicins) and *P. aeruginosa* (S-type pyocins) [46]. They are similar in size, structure, and function to colicins.

Phage-tail-like bacteriocins have structures similar to phage tails. They are cylindrical peptides with high molecular weights [23]. They are grouped into the R-type and F-type subclasses [23] (Figure 3).

R-type phage-tail-like bacteriocins bind to cell surface receptors, force the internal core into the microbial cell envelope, and determine rapid cell death [23]. They also affect macromolecule synthesis and oxygen uptake [9]. F-type phage-tail-like bacteriocins have a mechanism of action similar to R-type, but do not have contractile movement [27].

### 2.3. Fungal AMPs

**Figure 4:**
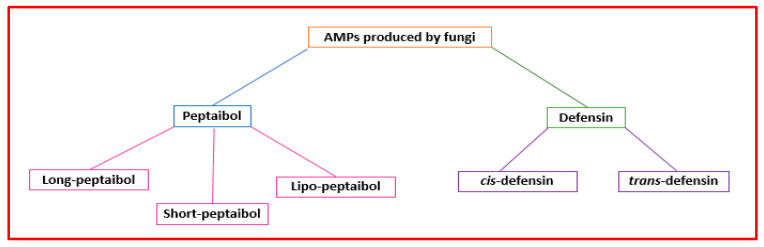
Classification of the AMPs produced by fungi.

Fungi produce peptaibols and fungal defensins [47,48] (Figure 4).

The term “peptaibol” is linked to structural characteristics. It is a combination of the words “peptide,” “*α*-aminoisobutyrate,” and “amino alcohol” [49]. Peptaibols are mainly made by *Trichoderma* fungi [50]. They are short peptides (containing 5–21 amino acids) with a high proportion of non-proteinogenic amino acids (i.e., α-aminoisobutyric acid), acylated *N*-terminal residue, and amino alcohol (i.e., leucenol or phenylalaninol) linked to the *C*-terminal [51]. Their three-dimensional structures consist of *α*-helix and *β*-bend patterns [52]. They are classified based on sequence length as “long” (18−20 residues), “short” (11−16 amino acids) (Figure 4), and founded on modification types on the terminal groups, “lipo” peptaibols (i.e., *N*-terminal acylated by decanoic) [53]. Different mechanisms have been proposed to describe their action. Concerning large peptaibols, it was hypothesized that their helical structures oligomerize and can form ion channels in the membrane. Instead, short peptaibols can form a pore via helical bundles (within the bilayer or by a barrel-stave mechanism) and interact with diverse molecular targets [9]. Peptaibols’ modes of action that do not involve interaction with the bacterial membrane include the inhibition of cell wall synthesis, DNA, protein synthesis, and that of relevant enzymes [10].

Eukaryotes and bacteria can produce defensins. Defensins are a class of cysteine-rich AMPs with short, cationic disulfide bridges [54]. They can be grouped into two superfamilies (cis and trans) (Figure 4). Fungi can produce *cis*-defensins with α-helical (cysteine-stabilized) or β-sheet folds. Defensins can disrupt the microbial cytoplasmic membrane, bind the bacterial precursor lipid II of the cell wall, or prevent cell wall biosynthesis [55].

### 2.4. Plant AMPs

**Figure 5:**
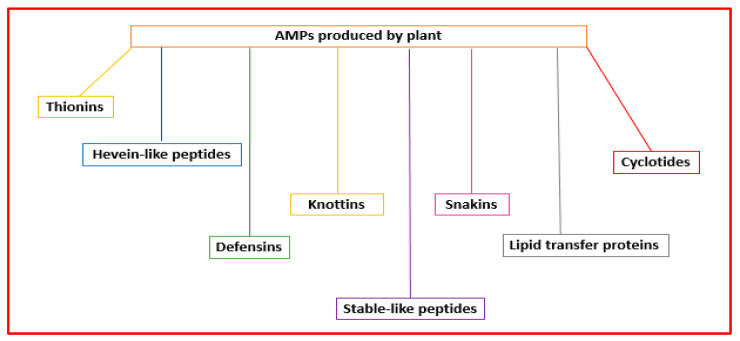
Classification of the AMPs produced by plants.

Plant AMPs are the first line of defense against infections produced by pathogenic microorganisms. They can have diverse structures and action mechanisms. Their classification is based on their tridimensional structures and amino acid sequence similarity, including thionins, hevein-like peptides, defensins, knottins, stable-like peptides, snakins, lipid transfer proteins, and cyclotides [56] (Figure 5).

#### 2.4.1. Thionins

Thionins are classified into five types indicated by Roman numerals, have sizes ranging from 45 to 48, and are found in monocots and dicots. They include two distinct superfamilies: α/β-thionins and γ-thionins [57]. α/β thionins have similar structures (homologous amino acid sequences) [58] and are rich in arginine, cysteine, and lysine. γ-thionins are similar to defensins, so some authors classify them in this group [59]. Thionins have a broad spectrum of activities. They act against Gram-positive and Gram-negative bacteria, yeast, fungi, insect larvae, and nematodes [60,61,62] and present cytotoxic effects against mammal cells in vitro [63].

#### 2.4.2. Hevein-like peptides

Hevein-like peptides can contain 29–45 amino acids with glycine (6), cysteine (8–10), and aromatic residues. They have a chitin-binding domain responsible for their antifungal activity [64] and 3–5 disulfide bonds that stabilize the antiparallel β-sheets and short α-helix [65]. The factors that favor chitin-binding are the three aromatic amino acids that give stability to the hydrophobic C-H group, the π electron system that determines van der Waals forces, and the hydrogen bonds between serine and N-acetylglucosamine [64]. Hevein-like peptides damage the fungal cell wall by interacting with hydrophobic residues and chitin present in the fungal cell [5]. They can constrain some enzymes’ activities by linking them with disulfide bonds [66].

#### 2.4.3. Defensins

Defensins can comprise 45–54 amino acids and four disulfide bridges. They have an antiparallel β sheet, are enclosed by an α-helix, and are limited by intramolecular disulfide bonds [67] called cysteine-stabilized αβ (CSαβ) motifs [68]. Defensins are resistant to proteolysis and are stable to variations in temperature and pH. They prevent microbial growth, trypsin, and α-amylase activities, decrease abiotic stress, and change the redox state of ascorbic acid [56].

#### 2.4.4. Knottins

Knottins, also called “cysteine-knot peptides”, are formed by 39 amino acids (of which six are cysteine residues), have three disulfide bonds (cysteine-knot motifs), and can be found in two conformations (cyclic and linear) [5,69]. They have high thermal stability and resistance to proteolytic action and can inhibit α-amylase, trypsin, carboxypeptidase, and cysteine protease [70,71]. They differ from protease inhibitors and defensins regarding cysteine space [5]. They are amphipathic peptides whose cationic portions can bind cell membranes, acid-sensing channels, and K^+^ and Na^+^ channels in membranes. Once they enter a cell, they attack intracellular targets (i.e., carboxypeptidases) and promote resistance [61]. Unfortunately, knottins are highly cytotoxic to human cells since their contact with membranes is not selective.

#### 2.4.5. Stable-like Peptides

Stable-like peptides are a class of small peptides that form a helix-loop-helix structure with a typical Cys motif of XnC1X3C2XnC3X3C4Xn (-X is an amino acid residue different from cysteine). Although their amino acid sequence is highly variable, the three-dimensional structure of stable-like peptides is conserved. They can have antifungal, antibacterial, ribosome-inactivating, and trypsin inhibiting activities [72]. Their bacteriostatic effect is due to binding with DNA, which decreases RNA and protein synthesis [73]. Their activity relates to the loop region that connects the two α-helices [74].

#### 2.4.6. Snakins

Snakins are generally small (~7 kDa), cysteine-rich, and positively charged proteins with antimicrobial, antinematode, and antifungal properties [75]. The mechanism of action is not precise. More than one hypothesis has been developed to explain it. Some authors believe they can promote immune responses by destabilizing the site of action through interaction with the negatively charged component [76,77]. Other authors hypothesized that they can act on phytohormone biosynthesis and transduction processes [78].

#### 2.4.7. Lipid Transfer Proteins

Lipid transfer proteins (LTPs) are small, cysteine-rich proteins (containing 100 aa) having 4 to 5 helices in their structure that are stabilized by hydrogen bonds. They can transfer lipids (i.e., fatty acids, phospholipids, acyl CoA fatty acids, and sterols) between membranes. In this way, they form pores and determine cell death. They can be classified into two subfamilies, LTP1 (relative molecular weight of 9  kDa) and LTP2 (relative molecular weight of 7 kDa), or into five types (LTP1, LTP2, LTPc, LTPd, and LTPg) based on the position of the conserved intron, the space between the cysteine residues, and the identity of the amino acid sequence [69].

#### 2.4.8. Cyclotides

Cyclotides are macrocyclic with cyclic cystine knot (CCK) structural motif peptides [79]. Disulfide bridges stabilize the head-to-tail cyclo. They can be classified into two subfamilies: Möbius and bracelets [80]. Their action depends on the cystine knot structural motif that promotes hydrophobic residue surface contact, some of which form a hydrophobic patch [81]. Cyclotides can act against bacteria, helminths, insects, and mollusks and have ecbolic anti-HIV and anticancer properties [81].

### 2.5. Animal AMPs

Vertebrate defensins are synthesized as “prepropeptides” and classified into α, β, and θ defensins [82]. They have short polypeptide sequences (18–45 amino acids), cationic net charges (+1 to +11), and three intramolecular disulfide bonds. In human α-defensins, the characteristic connections of disulfide bridges are Cys^1^–Cys^6^, Cys^2^–Cys^4^ and Cys^3^–Cys^5^ [83]. They are synthesized by promyelocytes and intestinal Paneth cells [84]. β-defensins differ from α-defensins in disulfide bond distributions and cysteine residues. The disulfide bridges in human β-defensins are Cys^1^–Cys^5^, Cys^2^–Cys^4^ and Cys^3^–Cys^6^ [83]. θ-defensins are cyclic octadecapeptides not expressed in humans and are active against *B. anthrax*, *S. aureus*, and *C. albicans* [85,86,87]. They contain a macrocyclic backbone and are structurally dissimilar to α- and β-defensins [88].

Invertebrates synthesize AMPs as components of humoral defense [89]. They are cationic peptides that can contain six or eight cysteine residues and show a cysteine-stabilized α/β motif [90]. The defensins produced by insects, arthropods, and mollusks contain six cysteines.

Eight cysteines form defensins made by mollusks and nematodes [91].

Invertebrate defensins are phylogenetically and structurally associated with vertebrate β-defensins. They have a hydrophobic domain (*N*-terminal) that can act against Gram-positive bacteria and a cationic domain (*C*-terminal containing six cysteines) that can act against Gram-negative bacteria [92].

Crustins (cationic cysteine-rich peptides that form a tightly packed structure) are found in crustaceans [93]. They have an *N*-terminal multidomain (rich in glycine, cysteine, and proline) and a *C*-terminal (with four *C*-terminal disulfide bridges) (Table 1) [94].

In fish, reptiles, amphibians, birds, and mammalians, AMPs ( size range of 15–200 residues) play an essential role in the immediate response to microorganisms [9]. Fish produce β-defensins, cathelicidins, hepicidins, histone-derived peptides, and piscidins [95] (Table 1).

Fish defensins are β-defensin-like proteins containing six cysteine motifs [96]. Cathelicidins are cationic proteins activated by elastase and other proteases discovered in the secretory granules of immune cells [97]. They act against Gram-positive and Gram-negative bacteria, parasites, fungi, and enveloped viruses [98,99,100,101,102]. Cathelicidins can bind and disrupt negatively charged membranes, alter RNA and DNA synthesis, damage the functions of enzymes and chaperones, and promote protein degradation [103].

Fish hepcidins are cysteine-rich peptides similar to human hepcidin with a hairpin structure linked via four disulfide bonds. They are iron-regulating antimicrobial hormones [104,105].

Hepcidins are grouped into HAMP1 and HAMP2 [95]. They act against bacteria (Gram-positive and Gram-negative) and fish pathogens and induce the internalization and degradation of ferroportin [106].

Piscidins are linear amphipathic AMPs. They have histidine residue and an α-helix that can interact with lipid bilayers [107]. They are classified into piscidins 1–7 based on their biological activity, amino acid sequence, and length [107].

Reptiles and avians produce cathelicidins and defensins (α-, β-, and θ-defensins) [108]. Cathelicidins are small-sized proteins made by macrophages and neutrophils [109].

Amphibians can produce magainin and cancrin (GSAQPYKQLHKVVNWDPYG) [13].

Mammalians make cathelicidin, defensin, platelet antimicrobial protein, dermcidin, and hepcidin AMPs [110]. Mammalian cathelicidins are cationic peptides with an amphipathic structure that assume α-helical, elongated conformations or β-hairpin forms [9].

## 3. Antimicrobial Peptide Structures and Activities

Most AMPs are made up of from 5 to 100 amino acids and have a positive net charge (generally lysine, arginine, and histidine amino acids; +2 to +11), with about 50% hydrophobic residues (generally aliphatic and aromatic amino acids) placed in variable sequence lengths [111,112]. AMPs can adapt to various structural changes when contacting microbe membranes [113]. Their amino acid compositions determine their charges, hydrophobic, and amphiphilic properties [114]. The number and quality of amino acids determine an AMP’s pharmacological applications. Generally, shorter AMPs are more antibacterial than long-chain linear peptides, which exhibit more hemolytic and cytotoxic activity [115]. Peptides with extremely short lengths have reduced antimicrobial potency since they have difficulty forming the amphipathic secondary structures responsible for the membrane-disruption capacity [116]. Generally, small amino acids, such as glycine, increase an AMP’s activity [117]. Glycine-rich peptides have high selectivity and antimicrobial ability (especially against Gram-negative bacteria) [118], as well as and antimycotic and anticancer activities [119,120].

Glycine-rich AMPs with net charges ranging from −1 to −2 that require cations as cofactors (i.e., Zn^2+^) have biocidal activity obtained by improving the eukaryotic innate immune response [121]. Proline-rich peptides can enter through membrane protein channels in the bacterial cytosol and modulate the immune system via angiogenesis or cytokine activity [122,123].

Cysteine-rich peptides can form pores in membranes [124]. The Cys residues can improve the AMP antimicrobial activity by stabilizing sheet or β-hairpin structures [125].

Aromatic-amino-acid-rich peptides cross the microbial membrane and disrupt it [126]. Trypsin-rich peptides stabilize the AMP tertiary structure since trypsin–trypsin interactions give a cross-strand contact [112].

Phenylalanine-rich peptides are highly hydrophobic molecules with intense antimicrobial activity against bacteria (Gram-positive and Gram-negative) and yeast [127]. They do not exhibit hemolytic activity [128].

Some lipopeptides (i.e., daptomycin, polymyxins B and E) and glycopeptides (i.e., teicoplanin, vancomycin, dalbavancin, telavancin, and oritavancin) are currently used for clinical purposes [129]. AMP secondary structures can be α-helices, β-sheets, non- α- or β- structures, or mixed structures [130]. Usually, amino acids with high helical propensity (i.e., alanine, arginine, leucine, lysine, etc.) synthesize novel antimicrobial peptides since α-helical structures promote interaction with membranes and determine membrane lysis [131,132,133]. Other characteristics that affect AMP activity are hydrophobicity and amphipathicity. AMPs with low hydrophobicity have antimicrobial activities since the self-association of peptides stops peptide passage through the cell wall [134]. Amphipathic AMPs have bactericidal and cytotoxic activities linked to their aptitude to form an α-helix [135]. They can interact with intracellular targets, damaging the membrane structure or making transient pores [134]. AMPs with high hydrophobicity have antimicrobial and hemolytic activities [136]. The high hydrophobicity of the α-helix improves the antimicrobial activity since the self-association of peptides stops peptide passage across the microbial cell wall [137] and enhances hemolytic activity, inducing peptides to penetrate deeper into the hydrophobic core of red blood cells [138]. In addition, amphipathic characteristics affect AMP activities. Imperfect amphiphilic peptides have more significant antimicrobial activity than perfect ones [139].

## 4. Antimicrobial Peptide Action

Mostly AMPs have a short half-life. They can act by disrupting the microbial membrane or without affecting membrane stability [9].

### 4.1. AMPs with Action on Cell Membranes

AMPs can make electrostatic interactions between their positive charges and the microbial cell surface’s negative ones, as well as hydrophobic relations between their amphipathic domain and the microbic membrane phospholipids [140]. The physical–chemical interactions and the interfacial properties determine the destabilization and permeabilization of the microbial membrane [8,141]. Both vertebrates and invertebrates produce AMPs (active in vitro at micromolar levels), which can affect the cell membrane by manipulating its components [142]. Gram-positive bacteria have a dense peptidoglycan layer, while Gram-negative ones have a fine peptidoglycan layer and an extra outer membrane [143]. Teichoic acid and lipopolysaccharides provide electronegative charges on the bacterial surface.

On the contrary, mammalian cell membranes do not have a net charge since the outer leaflet is formed by zwitterionic phospholipids (i.e., phosphatidylcholine, phosphatydylethanolamine, and sphingomyelin) [144] and the phospholipid bilayer is stabilized by cholesterol [145]. Thus, positively charged AMPs are significantly attracted by the negative charge (i.e., phospholipids, cardiolipin, phosphatidylglycerol, and phosphatidylserine) on bacterial membranes; instead, only weak hydrophobic interactions between AMPS and mammalian cell membranes can occur. Therefore, AMPs give selective antimicrobial effects without harming normal cells since the eukaryotic cell membranes have uncharged neutral residues (generally phospholipids, cholesterol, and sphingomyelins), which cannot interact with AMPs.

Highly cationic and anionic peptides have no antimicrobial activity [146,147].

Pore formation can be achieved by barrel-stave, toroidal pore [148], and carpet-like [149] mechanisms, the clustering of anionic lipids [150], aggregated channels [151], or more than one mechanism [9].

**Figure 6:**
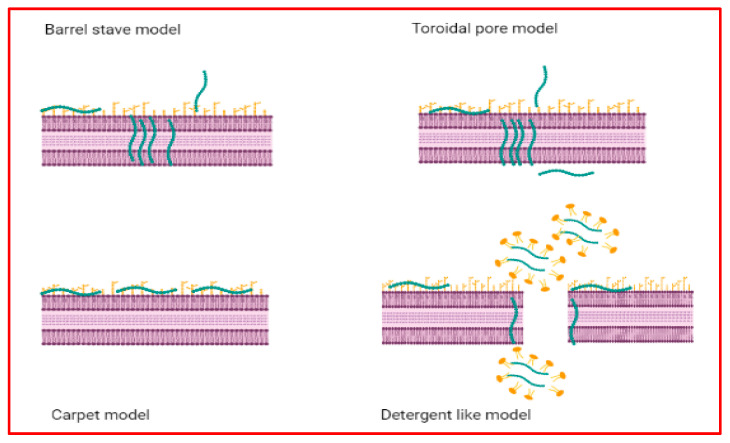
The AMP mechanisms of action on cell membranes.

The barrel-stave model hypothesizes that AMPs place themself alongside a membrane and penetrate the lipid bilayer. The pore external face is made by aligning the hydrophobic region of AMPs with the lipid bilayer’s central lipid region. Instead, the pore interior is made by the peptide hydrophilic contribution (by a peptide–peptide interaction) [152]. Barrel-like pores can determine cytoplasmic outflow, membrane collapse, and cell death [153] (Figure 6).

The toroidal pore model hypothesizes that AMPs vertically cross a lipid membrane without peptide–peptide interactions in the lipid membrane [152]. The pores are transient and less stable than barrel-stave formations [154] (Figure 6).

The carpet or detergent-like model assumes that AMPs are adsorbed parallel to the lipid membrane until wholly covered (like a carpet), inducing membrane disruption. In this process, no peptides across the membrane, peptide–peptide interactions, or peptide structures are made [155] (Figure 6). The AMP hydrophobic regions interact with the cell membrane, and the hydrophilicity ends with an aqueous solution [156].

Anionic lipid-clustering activity is obtained by forming phase-boundary defects between lipid domains due to interaction between cationic AMPs and anionic-charged lipids [150].

### 4.2. AMPs with No Action on Cell Membranes

Some AMPs can kill bacteria interfering with DNA (replication, transcription, and translation), cell division, and the blocking of protein biosynthesis and folding [113,157,158]. They can also interfere with the immune system, activating white blood cells, improving angiogenesis, blocking reactive oxygen and nitrogen species [159], suppressing toll-like receptors, reducing anti-endotoxin activity, interfering with cytokine-mediated production of cytokines [160], and influencing T- and B-cell activities [161]. Moreover, AMPs can bind cell membrane receptors (alternate ligand model) or affect receptor activation (membrane disruption model) by altering a receptor’s site or releasing a membrane-bound factor (transactivation model) that binds the receptor. Finally, AMPs can interfere with lipopolysaccharides, preventing inflammation [162].

## 5. AMP Potential in the Food Field

### 5.1. AMPs in Food Preservation

AMP application in food preservation is under review since they have a broad spectrum of activity (bacteria, fungi, and protozoa), good water solubility, and are thermostable, but the high cost of large-scale production limits their use [163,164]. AMP selection for food incorporation depends on their spectrum of activity and an AMP’s specificity toward microorganisms in a food product. For example, fermenticins produced by *Lactobacillus fermentum* [165] and defensins, which act on lipid II and lipid A at the bacterial membrane, show broad spectra of activity, pH, and temperature stability [166]. Some AMPs prolong the shelf-life of food by acting as antimicrobials and inhibiting lipid oxidation, such as peptides from *Cynoscion guatucupa* protein hydrolysate obtained by enzymatic hydrolysis with Alcalase and Protamex [167]. AMPs, stable at diverse ranges of pH levels, temperatures, and proteases, have been studied because, in food technology, temperature variations are used to increase the preservation of food, and proteases can be added to foods to decrease a food’s allergy power and alter its taste [168,169,170]. Tolerance to diverse pH conditions can be obtained by changing the sequence of AMPs. For example, adding histidine at the carboxyl terminus of a piscidin-like AMP allowed a more significant antimicrobial activity against *S. aureus* at pH 10.5 [171]. AMP stability can be improved by modifying an AMP’s geometrical properties (i.e., the radius of gyration, lipophilicity, ovality, polar surface area, and surface area). For example, the stability of Protegrin-1 was attributed to the high number of hydrogen bonds (distances <2.5 Å) [172]. The presence of free amino, sulfur, and carbonyl functional groups affected AMP bioactivity [173]. The concurrent addition of the additives ascorbate and nitrite could increase the carbonyl compounds in proteins, altering their functionality and technological properties [174]. Similarly, sulfites used as antioxidants and antiseptics could react with the disulfide bonds of AMPs to form irreversibly bound forms of S-sulfonates [175]. Thus, AMPs can be added to low-reactive foods such as fiber-rich food (whole-grain bread, cereals, pseudocereals, legumes, nuts, fruits, and vegetables) [176] and should not be inserted into high-reactivity food, such as liquid-based food formulations [177].

Nanoparticles, nanofibers, and nanoliposomes have been examined to protect AMP antimicrobial activity [178,179,180]. For example, nisin was placed into multifunctional soy-soluble polysaccharide-based nanocarriers to enhance its stability and preserve antioxidant and antimicrobial activity [181]. In raw and pasteurized milk, nisin-loaded chitosan/alginate nanoparticles were employed to prevent the growth of *S. aureus* during long incubation periods [182]. The nano-encapsulation of temporin B into chitosan nanoparticles enhanced the peptide’s antibacterial activity [183].

### 5.2. AMPs in Food Packaging

Active packaging systems have been developed to control the release of AMPs and decrease their interactions with food components. Pentocina MS1 and MS2 from *Lactobacillus pentosus* MS031 isolated from *Chinese Sichuan paocai* were added to fresh-cut fruits in cold packaging to decrease the growth of *Salmonella typhi*, *Listeria monocytogenes*, and *E. coli* [184]. Partially purified Gt2 peptides active against *E. coli* and *Salmonella typhi* were put into packages to preserve tomatoes [185]. The peptide MTP1 was employed in meat and dairy product packaging [186]. Nisin, which can inhibit the growth of *Listeria monocytogenes,* *Staphylococcus aureus, Penicillium* sp., and *Geotrichum* sp., was used to preserve mozzarella cheese. [187]. A fish protein hydrolysate was added to preserve fish flounder fillets [188]. Nisin preventing the growth of *Listeria monocytogenes* was employed to preserve cold-smoked salmon [189].

## 6. AMP Potential in the Pharmaceutical Field

### 6.1. AMP Antioxidant Potential

Some AMPs can act as free-radical scavengers, reduce lipid peroxidation, have metal ion chelation activity, and impact antioxidant enzyme activity (i.e., SOD, PPO, CAT, and GSH-Px) [190]. The presence of isoleucine, leucine, and histidine amino acids [191], as well as the number of active hydrogen sites, are essential for antioxidant activity [192].

### 6.2. Antineoplastic Agent

Currently, cancer is a leading cause of death worldwide. AMPs have some characteristics that make them potential drugs for cancer therapy, such as high activity, specificity and affinity, small size, slight drug–drug interaction, aptitude to cross membranes, and low toxic side effects since they do not accumulate in vital organs (i.e., the liver and kidneys) [193]. Moreover, they are easily modified and synthesized [194] and are less immunogenic than recombinant antibodies [195]. Therapeutic peptides are classified into three groups: antimicrobial or pore-forming peptides (anticancer peptides, or ACPs, naturally produced by all living creatures), cell-permeable peptides, and tumor-targeting peptides [195].

### 6.3. AMP Potential against Respiratory Diseases

Some natural and modified AMPs appear to have potential as drugs to cure respiratory diseases and as infection markers.

Pyocins, which can inhibit the growth of *P. aeruginosa*, could be used to cure fibrosis patients [196].

Esc (1−21)-c, a partial D-derivative of esculentin-1 that can decrease *P. aeruginosa* infection and has excellent resistance to degradation due to the elastase enzyme [197], could be employed to promote bronchial epithelium repair [198].

α-and β-defensins were potential infection markers of upper respiratory tract infection [199].

### 6.4. AMP Potential against Hypertension

Some AMPs (SAGGYIW and APATPSFW) could inhibit angiotensin-converting I (ACE), blocking the active site via weak interactions (i.e., electrostatic interaction, hydrogen bonds, and Van Der Waals interactions) [200]. ACE is an enzyme that can convert decapeptide angiotensin I (inactive) into octapeptide angiotensin II (vasoconstrictor), which is involved in hypertension and atherosclerosis [201].

### 6.5. AMP Potential against Obesity

EITPEKNPQLR, CQPHPGQTC, and RKQEEDEDEEQQRE are AMPs preventing pancreatic lipase activity. Pancreatic lipase is an enzyme that can hydrolyze 50–70% of food-derived fat in human organisms. Therefore, its inhibition is helpful in obesity treatment [202].

### 6.6. AMP Potential against Intestine Infection and Inflammation

α-defensins and C-type lectins (AMPs) are expressed in the gastrointestinal tract to sustain intestine symbiosis and protect it from pathological bacterial translocation [203].

### 6.7. AMP Potential against Viral Infections

Some AMPs can act against DNA and RNA viruses [204,205]. They can act on the viral envelope or after adsorption on the viral surface [206]. AMP positively charged residues can interact electrostatically with negatively charged cell surface molecules, such as heparan sulfate (glycosaminoglycans) [207], prevent the spread across tight junctions of the virus from one cell to another cell (cell-to-cell spread), or prevent the formation of giant cells (syncytium) [13].

Lactoferrin (iron-binding glycoprotein) can act as an antiviral material by inhibiting the replication of a wide range of DNA and RNA viruses or preventing virus entry into a host cell through direct binding to virus particles or blocking cellular receptors [208].

Defensins (α- and β-) can act against human immunodeficiency virus (HIV), influenza, herpes simplex virus (HSV), and SARS-CoV [209]. It has also been hypothesized that an infusion of defensins during *Cytomegalovirus* infections may be helpful in the treatment of COVID-19 in pregnant women [210,211].

Frog-skin-derived peptide AR-23 and some of its derivatives can act against the viral surfaces of all enveloped viruses (i.e., coronaviruses, including SARS-CoV-2; paramyxoviruses; and herpesvirus) [212,213].

### 6.8. AMP Potential against Skin Infections

AMPs can be considered as a therapeutic option since they have a broad spectrum of biological activities against microbes; remain on an application site when topically administrated; and support wound healing by controlling angiogenesis, cell migration, and cytokine release chemotaxis [214]. Human keratinocytes and the granular skin layer make and store AMPs and lipids within secretory granules (lamellar bodies) [215]. The lamellar bodies make a physical barrier in superficial layers of the epidermis that can inhibit microbial growth and water loss. RNase 5 and RNase 7 are AMPs present in healthy human skin. They are active on Gram-negative and Gram-positive bacteria [216]. Other AMPs involved in skin wellbeing are psoriasin; calprotectin (iron- and zinc-binding S100 proteins) expressed by keratinocytes; β-defensins; the cathelicidin hCAP18, which must be converted to the active form; LL-37; histone 4 (active against Gram-positive bacteria) and dermcidin (active against antibacterial and antifungal mechanisms) produced by pilosebaceous follicles and eccrine glands, respectively; and α-defensins and LL-37 formed by neutrophils and natural killer cells [217]. Bee venom peptides can be helpful as a topical agent to promote skin regeneration and acne treatment. [218,219,220].

## 7. Conclusions

This work summarized the current knowledge regarding antimicrobial biopeptides to highlight their potential applications in the industrial field. Researchers are examining new sources of bioactive materials to use as natural preservatives in foods and to reduce the emergence of antibiotic drug resistance. AMPs seem to have good prospects as natural preservatives incorporated in food and food packaging, as well as for antioxidant, antineoplastic, antiobesity, antihypertensive, anti-inflammatory, antiviral, and dermatological agent drugs. Nanocarriers can be used to improve their bioavailability. Nevertheless, large-scale production and high cost of production could limit their use.

## Figures and Tables

**Table 1:** Animal AMPs.

Animals	AMPs
Mammalians	cathelicidins defensins (α-, β-, and θ-defensins; θ-defensins are not expressed in adult humans) platelet antimicrobial proteins dermicidins hepcidins
Reptiles	defensins (α, β-, and θ-defensins) cathelicidins
Fish	β-defensins cathelicidins hepicidins (HAMP1 and HAMP2) histone-derived peptides piscidins (piscidins 1–7)
Amphibians	magainins cancrins
Crustaceans	crustins

## Data Availability

Not applicable.
